# NOX as a Therapeutic Target in Liver Disease

**DOI:** 10.3390/antiox11102038

**Published:** 2022-10-16

**Authors:** Deyamira Matuz-Mares, Héctor Vázquez-Meza, María Magdalena Vilchis-Landeros

**Affiliations:** Departamento de Bioquímica, Facultad de Medicina, Universidad Nacional Autónoma de México, Coyoacán, Ciudad de México CP 04510, Mexico

**Keywords:** NOX, fibrosis, liver, cancer, ROS

## Abstract

The nicotinamide adenine dinucleotide phosphate hydrogen oxidase (NADPH oxidase or NOX) plays a critical role in the inflammatory response and fibrosis in several organs such as the lungs, pancreas, kidney, liver, and heart. In the liver, NOXs contribute, through the generation of reactive oxygen species (ROS), to hepatic fibrosis by acting through multiple pathways, including hepatic stellate cell activation, proliferation, survival, and migration of hepatic stellate cells; hepatocyte apoptosis, enhancement of fibrogenic mediators, and mediation of an inflammatory cascade in both Kupffer cells and hepatic stellate cells. ROS are overwhelmingly produced during malignant transformation and hepatic carcinogenesis (HCC), creating an oxidative microenvironment that can cause different and various types of cellular stress, including DNA damage, ER stress, cell death of damaged hepatocytes, and oxidative stress. NOX1, NOX2, and NOX4, members of the NADPH oxidase family, have been linked to the production of ROS in the liver. This review will analyze some diseases related to an increase in oxidative stress and its relationship with the NOX family, as well as discuss some therapies proposed to slow down or control the disease’s progression.

## 1. Introduction

Chronic liver diseases have high rates of morbidity and mortality around the world. Each year, 2 million people die from these diseases worldwide [1]. These illnesses include disorders that result in disease when liver function is disturbed. The main causes of chronic liver disorders cirrhosis are chronic hepatitis B virus (HBV), hepatitis C virus (HCV), alcohol-related liver disease (ALD), and nonalcoholic fatty liver disease (NAFLD). All these causes produce an excess of ROS [2].

All aerobic organisms generate ROS; they are regulated by cellular metabolism and by antioxidant defenses, whether enzymatic (catalase, SOD, peroxidases) or non-enzymatic (glutathione, vitamins C and E) [3,4,5]. However, when there is an imbalance between the ROS production and antioxidant defenses, a state of oxidative stress is generated, which causes important metabolic changes in the cell [6]. If the oxidative stress in the cell is not controlled or diminished, serious metabolic and neuronal disorders can occur, as well as cell death [7,8,9].

**Figure 1:**
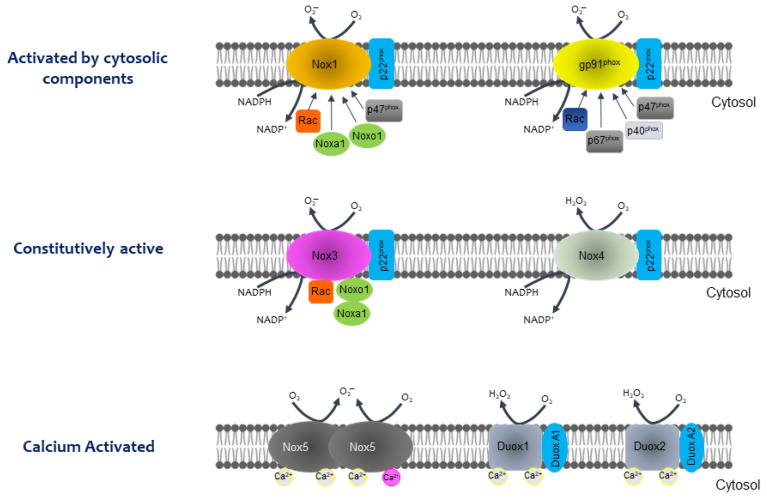
Proposed models for isoforms of the NOX family (Modified from [11]).

A family of oxidases, dependent on NADPH, is responsible for the production of ROS that regulates various cellular metabolic activities. This family is made up of seven members and divided into two groups: five NADPH oxidases (1–5), known as NOX, and two dual oxidases (DUOX1 and 2), the latter calcium-dependent (Figure 1) [10,11]. The NOX family, involved in the host defense system, consists of transmembrane proteins which transport electrons through biological membranes to reduce oxygen to superoxide anion (O_2_^●−^) [12].

## 2. The NOX Family: From the Respiratory Burst to the Regulation of Metabolic Pathways

In the 1930s, the first studies were carried out on phagocytes (including neutrophils, macrophages, and eosinophils) in which it was determined that there were changes in oxygen consumption and that these modifications were the product of different stimuli, for example, infection by microorganisms. Within the changes in oxygen consumption, it has been determined that there was excessive consumption of O_2_, which significantly increases ROS production (such as H_2_O_2_ and O_2_^●−^). It has been described that O_2_ consumption is not only used for cellular respiration, but also to generate large quantities of highly reactive molecules with microbicidal activity such as hypochlorite [13].

In addition, in the phagocyte, there is excessive consumption of glucose via the pentose phosphate pathway (such as glucose 6-P) for the generation of NADPH, which is used or consumed by these cells. This process, known as respiratory burst, is carried out by NOX, which has a catalytic core consisting of 2 membrane-bound subunits (gp91^phox^ and p22^phox^) and cytosolic components (p47^phox^, p67^phox^, and p40^phox^). Another cytosolic component, a small G protein called Rac (Rac1 in non-phagocytic cells and Rac2 in phagocytic cells), is also necessary to fully activate this NADPH-dependent complex [13].

There are other components of the same family of oxidases that have different functions besides generating ROS. As described in Figure 1, the NOX family can be divided into three large groups: (i) those activated by cytosolic components (NOX1 and NOX2); (ii) those that are constitutively activated (NOX3 and NOX4) and (iii) those that are dependent or activated by calcium (NOX5, DUOX1, and DUOX2) [11]. 

On the other hand, NOX and DUOX have characteristics that make them belong to this family of oxidases:

1. They are dependent on NADPH;
2. They are only found in membrane systems (plasma, mitochondrial, etc.);
3. NOX consists of a catalytic subunit (gp96^phox^-like) linked to another subunit (p22^phox^), in most cases, and some subunits that regulate the activity of this enzyme system (p47^phox^, p67^phox^, p40^phox^, and Rac). The catalytic subunit has six transmembrane domains (seven for DUOX1 and 2), four hemes in transmembrane domains three and five, an NADPH-binding domain, and a FAD-binding domain at the C-terminus in the cytosolic region.

The route followed by the electrons that are used for the formation of O_2_^●−^ is as follows: they are transferred from NADPH in the cytosol, to FAD in the dehydrogenase domain, then to the internal and external haem in the transmembrane domain, and finally, to O_2_ outside the cell, producing O_2_^●−^. The general reaction is shown below:

NADPH + 2O_2_ ⇔ NADP^+^ + 2O_2_^●^^−^ + H^+^

The O_2_^●−^ generated in these membrane proteins and their derivatives (other ROS) can affect cells, organs, or tissues when there are insufficient antioxidant systems to control or cancel them, generating oxidative stress. In addition, when ROS production is prolonged by a redox imbalance, it can be related to changes in the cells and affect some cellular pathways, such as cell development and differentiation, hormone biosynthesis, cell aging, apoptosis, responses to oxygen changes (oxygen sensing), growth factors, hormones, and cytokines, which, over the time, aggravate a person’s health [14]. 

Many mechanisms regulate NOX activity, including calcium, free fatty acids, protein–protein interactions, intracellular trafficking, and post-translational changes such as phosphorylation or acetylation [11]. Due to this, it has been concluded that the regulation of NOX activity is very complex. Furthermore, depending on the state of cell activity, NOX is selectively activated or deactivated.

The complex signaling aspects upstream of these events make it plausible to develop NOX inhibitors, which selectively attenuate disease-related NOX-mediated ROS formation without altering physiological ROS signaling.

NOX plays a crucial role in the inflammatory response and fibrosis in several organs, such as the lungs [15], pancreas [16], kidney [17], liver [18,19] and heart [20]. NOX has a wide range of physiological roles, including cellular growth, serotonin biosynthesis, endothelial signaling, control of renal processes, and the immune response to pathogens (as a source of the so-called oxidative burst). However, its overexpression is linked to many neurological disorders and cancer types [21,22,23].

In this review, we will focus on analyzing some diseases related to an increase in oxidative stress, the latter’s relationship with the NOX family, some therapies proposed to stop the progression of the related diseases and highlight successful therapies. 

## 3. Cellular Distribution of NOX in the Liver

The liver is a key organ in the body and is the central metabolic coordinator. This organ has two main lobes. Each lobe has eight segments. Segments are composed of hexagonal lobes with portal triads (portal vein, bile duct, and hepatic artery) and hepatocytes arranged in linear cords radiating from a central vein [24]. The hepatic artery supplies oxygen and the hepatic portal vein supplies nutrients. The hepatocytes are heterogeneous and have different metabolic functions depending on their proximity to central veins or portal veins (spatial zonation). This phenomenon explains how the liver handles opposing metabolic functions. Pericentral hepatocytes carry out glutamine synthesis, glycolysis, lipogenesis, bile acid synthesis, and xenobiotic metabolism. On the other hand, periportal hepatocytes are more active in gluconeogenesis, ureagenesis, cholesterol biosynthesis, fatty acid oxidation, and protein secretion. Middle-lobe hepatocytes specialize in iron homeostasis, among other functions [25]. 

In addition to hepatocytes, the liver contains nonparenchymal liver cells that are important in maintaining liver structure and function. These include hepatic stellate cells (HSCs) involved in extracellular matrix biosynthesis following liver injury, and endothelial cells (ECs), including hepatic sinusoidal ECs (LSECs), vascular ECs, and lymphatic ECs (LyECs), which play a key role in liver homeostasis, regulating intrahepatic vascular tone, immune cell function, and hepatic stellate cell (HSC) quiescence [24,26,27]. Other nonparenchymal cells include Kupffer cells (KC, resident macrophages), T cells, and dendritic cells, all of which contribute to the immune response [28]. Nonparenchymal cells are also exposed to microenvironments created by the factor gradient across the lobule. These gradients probably also modulate their gene expression, morphology, and function [29].

**Figure 2:**
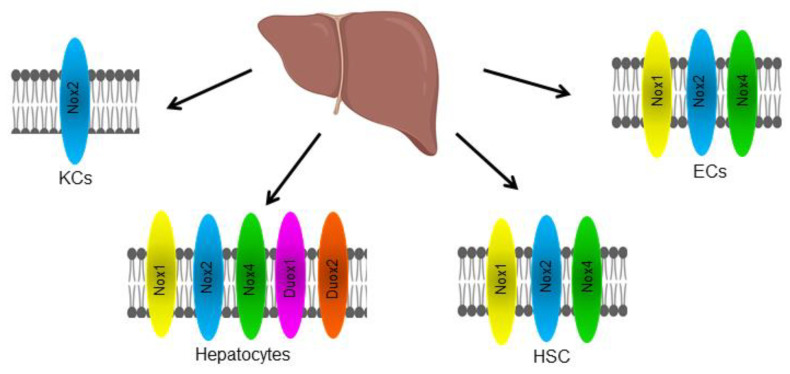
Cellular distribution of isoforms of the NOX family in the liver. Liver cells, such as hepatocytes, Kupffer cells (KCs), endothelial cells (ECs), and hepatic stellate cells (HSCs), express different NOX isoforms.

Different liver cell types, including hepatocytes, hepatic stellate cells (HSCs), Kupffer cells (KCs), endothelial cells (ECs), and infiltrating leukocytes, express NOX isoforms differently (Figure 2). NOX1, NOX2, NOX4, DUOX1, and DUOX2 are expressed by hepatocytes; NOX2 is expressed by KCs, which are resident liver macrophages; NOX1, NOX2, and NOX4 are expressed by HSCs; and NOX1, NOX2, and NOX4 are also expressed by ECs [12].

Our research group found the expression of NOX1, 2, and 4 in rat hepatocytes isolated by collagen perfusion. In these cells, only NOX2 participated in the regulation of metabolic pathways such as gluconeogenesis, glycogenolysis, and ureagenesis activated by adrenaline [30,31]. The hepatocytes that are carrying out these metabolic pathways would correspond to the periportal hepatocytes, as mentioned above.

These NOX contribute to liver fibrosis by acting through multiple pathways, including HSC activation, proliferation, survival, and migration; hepatocyte apoptosis, enhancement of fibrogenic mediators, and the mediation of an inflammatory cascade in both KCs and HSCs [32].

## 4. NOXs and Inflammasomes Activation

One of the vascular tissue responses to damage-causing stimuli is inflammation which initiates the healing and repair process [33]. Inflammation is regulated by complexes of macromolecules known as inflammasomes. They are made up of a sensor protein, which is a pattern recognition receptor (PRR) that forms oligomers in response to damage-associated molecular patterns (DAMPs), pathogen-associated molecular patterns (PAMPs), or homeostasis-altering molecular processes (HAMPs) [33]. 

The most studied PRRs are the NOD-type receptors formed by proteins with oligomerization domain and nucleotide binding, and a leucine-rich repeat domain (NLR). This family of receptors consists of three subfamilies: nucleotide-binding oligomerization domain (NOD), NOD-like receptor CARD domain-containing (NLRC), and NOD-like receptor pyrin domain-containing (NLRP). NLRP is most closely related to the inflammasome. Fourteen different NLRPs are activated by exogenous signals via PAMPs or by endogenous signals via DAMPs [1,2,3,34,35,36]. The most extensively researched inflammasome, NLRP3, has been related to the occurrence of chronic inflammation, and neurological and metabolic illnesses, including fibrosis and pathologies of the liver [37].

**Figure 3:**
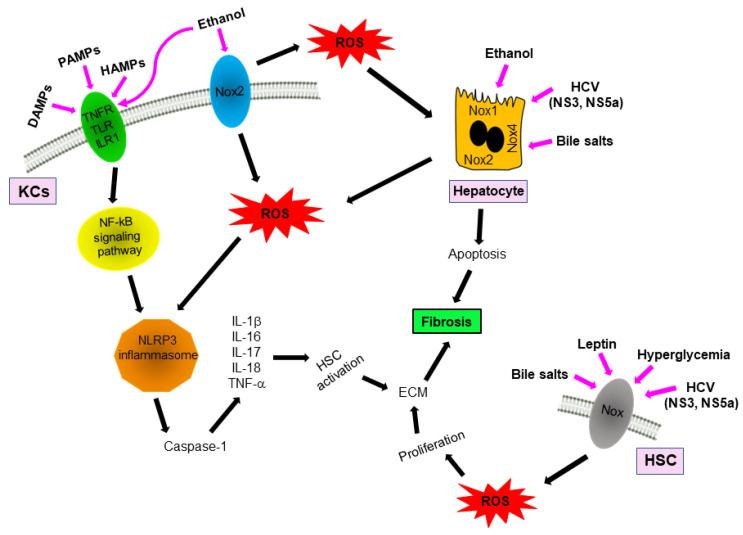
Fibrosis process in the liver with the participation of NOX. Tissue response to damage-causing stimuli is regulated by inflammasomes (NLRP3). NLRP3 are activated by damage-associated molecular patterns (DAMPs), pathogen-associated molecular patterns (PAMPs), or homeostasis-altering molecular processes (HAMPs). Oxidative stress is crucial in this process. KCs and damaged hepatocytes in the liver release a large amount of ROS. Tumor necrosis factor receptor (TNFR), Toll-like receptors (TLR), and IL receptor 1 (ILR1) activation cause signaling of NLRP3 through NFκB. In KCs, NLRP3 participates in caspase-1 activation. Caspase-1 controls the release of IL-1, IL-16, IL-17, IL-18, and TNF-α. These cytokines cause HSC activation with the deposition of extracellular matrix (ECM) and liver fibrosis. Various stimuli such as ethanol, bile salts, HCV, leptin, and hyperglycemia activate NOX in different liver cells, producing ROS and fibrosis.

Oxidative stress is essential for the assembly and activation of NLRP3 inflammasome [38]. In reaction to toxic compounds, KCs in the liver release a large amount of ROS [39], and damaged hepatocytes may also release ROS and DAMPs [40]. DAMPs cause the activation of the tumor necrosis factor receptor (TNFR), Toll-like receptors (TLR), and IL receptor 1, which in turn causes the signaling of the NLRP3 inflammasome [41,42,43]. Additionally, transcription of pro-IL-1, pro-IL-18, NLRP3, and interferon genes is induced by the binding of MyD 88 to TLR4 and translocation of NF-κB to the nucleus [44]. On the other hand, although some data show that NOX does not activate inflammasomes, they suggest that ROS production by NLRP3 activators involves NOX [45]. NOX-produced ROS causes NLRP3 to assemble with CARD-containing adapter protein (ASC) and recruit pro-caspase 1 [46]. Furthermore, NOX inhibitors such as DPI or (2R,4R)-4-aminopyrrolidine-2,4-dicarboxylate inhibit NLRP3 inflammasome activation [47]. Indeed, DPI inhibits caspase-1-mediated IL-18 activation in mice undergoing physical stress [48]. When the NLRP3 inflammasome and ROS are activated in KCs, caspase-1 is also activated. Caspase-1 controls the maturation and release of IL-1, which exacerbates inflammation. The increase in IL-16 and IL-17 mediated by NF-κB, and the production of IL-1β through the NLRP3 inflammasome, triggers the activation of HSCs with the deposition of a greater amount of extracellular matrix (ECM), which produces liver fibrosis [49,50] (Figure 3). Thus, pro-inflammatory caspase-1 is proteolytically cleaved and activated by inflammasome activation, which leads to the release of pro-inflammatory cytokines and causes cell death. 

## 5. NOXs and Fibrosis

Fibrosis is caused by the accumulation of ECM proteins, mainly type I collagen. HSCs (Ito cells) in the liver are the main collagen-producing cells [51]. In chronic liver disorders, these cell types undergo phenotypic changes. Quiescent HSCs transform into myofibroblasts in the damaged liver, producing inflammatory cytokines and many ECM proteins, including collagen, fibronectin, nodulin, elastin, laminin, entactin, tenascin, hyaluronan, and other proteoglycans [52]. In addition to HSCs, portal fibroblasts and bone marrow cells have fibrogenic potential [53,54]. 

NOX is the primary generating source of ROS in HSCs and KCs. Isoforms of NOX that have an essential role in the activation of HSC and liver fibrogenesis are NOX1, NOX2, and NOX4 [55,56]. Additionally, an area of the promoter of collagen genes sensitive to ROS and H_2_O_2_ has been described [57]. In rat HSCs, transforming growth factor β1 (TGF-β1) stimulates the production of α1(I)Procollagen mRNA through an H_2_O_2_-C/EBPb-dependent mechanism. TGF-β induces H_2_O_2_ accumulation, and this oxidant up-regulates col1α1 gene expression. The TGF-β induces binding of a CCAAT/enhancer binding protein-b(C/EBPb) transcriptional complex between nucleotides 2370 to 2344 of the col1a1 promoter. These actions are mimicked by the administration of H_2_O_2_ and abrogated by the addition of catalase to the cultured cells. These data suggest that H_2_O_2_ is an important mediator in TGF-b-elicited col1a1 up-regulation in HSCs [58]. The imbalance between the generation of ROS and clearance of these reactive substances causes oxidative stress, a crucial process in the pathogenesis of liver fibrosis [59].

HSCs express NOX2 and are activated by apoptotic hepatocytes that trigger the synthesis of α-smooth muscle actin (αSMA), collagen I, and TGF-β [60]. NOX2-derived ROS could activate collagen I transcription in HSCs. In response to CCl_4_ injection or bile duct ligation (BDL), NOX2^−/−^ mice have reduced fibrosis. However, NOX1 levels are elevated in fibrotic livers and active HSCs, and NOX1^−/−^ mice have reduced fibrosis in response to CCl_4_ or BDL injection [61]. In p47^phox−/−^ mice, angiotensin II is also a significant inducer of NOX-mediated ROS generation, and liver fibrosis, and the expression of procollagen 1 (I), TGF-β, and Timp1 are attenuated in p47^phox−/−^ mice [62]. NOX4 mediates the effects of TGF-β, such as the death of hepatocytes and the activation of HSCs into myofibroblasts. Both NOX4^−/−^ and hepatocyte-specific deletion of NOX4 (Nox4hepKO) mice exhibit fast recovery and increased survival following liver regeneration after a 2/3 partial hepatectomy [63].

The main causes of liver fibrosis are chronic hepatitis C virus (HCV) or hepatitis B virus (HBV) infection, non-alcohol-associated steatohepatitis (NASH), and alcohol consumption [64].

## 6. Alcohol and Non-Alcohol Associated Steatohepatitis

Oxidative stress is crucial in alcohol and non-alcohol-associated steatohepatitis (ASH and NASH) [65]. Activation of NF-κB by NOX2 in KC is crucial in the pathophysiology of early alcohol-induced hepatitis, activating the production of cytotoxic TNF-α [66]. Apoptosis of hepatocytes is caused by ethanol-induced oxidative stress and produces hepatic fibrosis by releasing profibrogenic cytokines and HSC activation [67]. There is evidence that ethanol induces ROS by NOX. Chronic ethanol feeding increased LPS-stimulated NADPH oxidase-dependent ROS production in KCs, causing an increase in TNF-α [68]. The p47^phox^ subunit appears to significantly impact alcohol-associated steatohepatitis through a mechanism involving lipid metabolism proteins [69]. On the other hand, NOX and hypoxia inducible factor 1α (HIF-1α) were shown to be involved in alcohol-mediated induction of endothelin-1 (ET-1) in liver sinusoidal endothelial cells (LSEC). This effect was attenuated by p47^phox^ siRNA transfection, suggesting NOX1 or NOX2 activation in these cells [70]. When alcohol is intragastrically administered to p47^phox^-deficient mice, less liver damage and hepatic free radical generation were observed compared to wild-type mice [71]. Early ethanol-induced liver damage causes KCs to express NOX2 and produce ROS [71]. These findings suggest that ROS produced by NOX in KCs are crucial to the pathophysiology of alcohol-induced hepatitis. Analysis of hepatic gene expression in alcohol-associated hepatitis patients demonstrates the significance of NOX in the etiology of the disease. In human livers with alcohol-associated hepatitis, the expression of genes encoding ECM, such as procollagen 1(I), fibrogenesis mediators, and inflammatory cytokines is markedly increased. In addition, several NOX isoforms, or components, including NOX4, p22^phox^, Rac1, DUOX1, and DUOX2, are increased in livers with alcohol-associated hepatitis compared with normal livers [72].

Among the most potent risk factors for NASH leading to liver fibrosis and cirrhosis are metabolic syndrome and diabetes mellitus. Persistent hyperglycemia can stimulate HSC proliferation and ECM production through NOX activation [73]. The rise in leptin-mediated NOX activity in HSCs is an important factor in fibrosis progression in NASH [74]. Leptin induces collagen 1α(I) through a NOX-mediated pathway [75]. Advanced glycation end products (AGE) that build up in diabetic patients cause tissue damage by activating AGE receptors (RAGE) and releasing ROS [76]. Furthermore, NOX4 activation in hepatocytes is proapoptotic under different conditions and is likely to contribute to the progression of NASH [77]. It is also important to note that NOX could confer individual susceptibility to metabolic syndrome and NASH by polymorphisms. Polymorphism in the promoter region of the NOX4 gene was recently shown to be associated with increased caloric intake and ROS levels in peripheral blood mononuclear cells [78].

## 7. Hepatitis C Virus (HCV) Induced Hepatocellular Damage

The hepatitis C virus (HCV) is a global health concern because the infection often leads to chronic hepatitis C that eventually progresses to liver cirrhosis and liver cancer [79]. There is evidence indicating that the progression and development of chronic hepatitis C depend on ROS [80]. Among the HCV proteins, the core protein, NS3, and NS5a are associated with heightened oxidative stress [81,82]. In human monocytes, recombinant NS3 protein phosphorylates p47^phox^ activates NOX, and produces O_2_^●−^ [81]. Increased ROS production, steatosis, and cell transformation caused by HCV protein expression in hepatocytes lead to liver cancer [4,83]. TGF-β induces NOX4 in HSCs and hepatocytes [84,85]. The HCV core protein upregulates TGF-β mRNA in HepG2 cells, and treatment of cells with a TGF-β blocking antibody prevents HCV-mediated NOX4 induction and reduces O_2_^●−^ production [86]. Moreover, HCV core and NS3 proteins increase ROS production in human HSCs. TGF-β1 secretion and type I collagen expression in HSCs are increased by HCV core and NS3–NS5 proteins [87]. Thus, at least in part, ROS produced by NOX is the way HCV causes liver fibrosis and inflammation.

However, it is worth noting that more than 90% of hepatitis C virus-infected patients are treated with direct-acting antiviral agents (DAAs) that prevent the progression of liver disease and decrease the elevation of hepatocellular carcinoma (HCC) [79]. The identification of the non-structural proteins (p7, NS2, NS3, NS4A, NS4B, NS5A, and NS5B) along with a better understanding of their roles in the viral life cycle has been the key breakthrough for the development of DAAs. This knowledge has led to the identification of substances that block crucial steps in the HCV replication cycle. There are currently three main classes of HCV antiviral drugs: inhibitors of the NS3/NS4A protease (PIs) [88], inhibitors of the NS5A complex [89], and inhibitors of the NS5B polymerase, which are further sub-divided into nucleos(t)ide (NI) and non-nucleos(t)ide (NNI) inhibitors [90]. Above all, combinations of two or three of these agents have been shown to be highly effective in inducing a sustained virologic response (SVR), with persistent loss of HCV RNA from serum [91].

Although DAA offers a safe and effective therapy for chronic HCV patients, some challenges must be considered: the presence of resistant variations, low efficacy in cirrhotic patients, the presence of drug interactions, and cost. Continuous monitoring and use of combined groups of DAAs with different mechanisms of action should be carried out to minimize resistance, as well as the search for other antiviral groups with different mechanisms of action, and the development of antifibrotic drugs to improve cirrhosis. In addition, even though direct-acting antivirals (DAAs) offer high cure rates in people with hepatitis C virus (HCV) infection, and treatment is shorter than with other drugs (8 to 12 weeks), DAAs are very expensive. Their average cash prices typically begin at over USD 10,000 for one month’s supply. In some countries, many people can pay less by using their insurance and/or various aid programs. It is probable that many people cannot access these drugs due to their cost [92]. 

## 8. Hydrophobic Bile Salts-Induced Liver Injury

Biliary lipids consist mainly of bile salts, phospholipids, and cholesterol, which form mixed micelles and vesicles. Bile salts play various physiological roles but have damaging effects on cell membranes due to their detergent properties since they can disrupt cellular membranes, which causes cholestasis and hepatocellular injury [93,94]. They can also promote the generation of ROS that, in turn, oxidatively modify lipids, proteins, and nucleic acids, and eventually cause hepatocyte necrosis and apoptosis [95]. Besides, by interacting with receptors and activating signaling pathways, they participate in a diverse set of regulatory processes [96]. In particular, through the activation of the nuclear farnesoid receptor (FXR or NR1H4) [97,98], they have a role in the regulation of their own synthesis, and thereby, in cholesterol and whole-body lipid homeostasis [99], as well as in the control of glucose and energy metabolism [100]. Additionally, by being a ligand for the G-protein coupled receptor TGR5 [101] and activating mitogen-activated protein kinase pathways [102], they contribute to several additional cell signaling and immunoregulatory processes [103,104].

As already mentioned, bile acids are end products of cholesterol catabolism and are critical for the normal absorption of cholesterol, lipids, and fat-soluble vitamins in the intestine. However, because of the intrinsic toxicity of bile acids, their levels need to be strictly regulated. By regulating the expression of genes involved in bile acid synthesis, conjugation, and transportation, FXR turns out to be the main regulator of bile acid homeostasis [105].

FXR is a member of the nuclear hormone receptor superfamily. Members of this superfamily regulate various physiological processes, including development, differentiation, metabolism, and homeostasis [106]. FXR is highly expressed in the liver, intestine, kidney, and adrenal gland. The identification of bile acids as bona fide FXR endogenous ligands reveals an essential function of FXR in controlling bile acid metabolism [107]. A large body of evidence indicates that the major function of FXR is to control bile acid homeostasis and to prevent bile acid-induced liver toxicity [108]. Moreover, it also plays an important role in regulating liver regeneration [5,6,106,109], hepatic fibrosis [110], cholestasis [111], and hepatic inflammation [112]. 

FXR activity is a major inhibitor of HCC. Whole-body FXR-deficient mice spontaneously develop liver tumors [113] in which the activation of the Wnt/β-catenin signaling pathway and oxidative stress were identified as the major drivers [114]. Nevertheless, liver-specific FXR deficiency in mice does not induce spontaneous liver tumorigenesis but may only serve as a tumor initiator [115]. The persistently high levels of bile acid enhanced inflammation and bile duct proliferation and led to the downregulation of FXR expression. Those data indicate that during hepatocarcinogenesis, bile acid may function as a tumor promoter as well as a DNA damage initiator [116,117]. 

On the other hand, hydrophobic bile salts activate NOX through a ceramide and protein kinase C-dependent pathway as an important upstream event of bile salt-induced hepatocyte apoptosis [118]. Hepatocytes can undergo apoptosis via CD95 when exposed to hydrophobic bile salts. Protein kinase C f (PKCf) is activated by taurolithocholate-3-sulfate, which also encourages the synthesis of ceramides and causes acidic sphingomyelinase to become active [119]. Activated PKCf induces serine phosphorylation of p47^phox^ [120]. NOX-derived ROS activate JNK and an Src family kinase, activating epidermal growth factor receptor (EGFR) [121]. This activation of EGFR causes CD95 tyrosine phosphorylation, the creation of the death-inducing signaling complex, and hepatocyte apoptosis [122]. In contrast to hepatocytes, in HSCs, bile acid-induced EGFR activation promotes cell proliferation rather than death [123]. This may be explained by the fact that hydrophobic bile acids fail to induce JNK activation in HSC despite the induction of a NOX-driven ROS response, which triggers JNK activation in hepatocytes, but not in HSC [120].

## 9. Role of ROS in Acute and Chronic Liver Damage

Even though the pathogenesis of liver fibrosis differs depending on the cause of the damage, oxidative stress appears to be a key factor in most liver fibrogenesis types [124]. The liver damage is manifested mainly with the death of hepatocytes. When the damage is limited, for example after acute hepatitis, a regenerative response of hepatocytes occurs, replacing the affected tissue and restoring the normal hepatic architecture [125]. During rapid liver regeneration, hydrogen peroxide contributes to cell proliferation, differentiation, and angiogenesis. For the best liver repair, neutrophils direct inflammatory monocytes and macrophages to take on a pro-regenerative phenotype, possibly through ROS [126]. Hydrogen peroxide also modulates the proliferation/quiescence switch in the liver post-hepatectomy regeneration. Extracellular signal-regulated kinase (ERK) signaling in hepatocytes must be activated by high H_2_O_2_ levels to cause a transition from quiescence to proliferation. On the other hand, sustained low H_2_O_2_ levels are necessary to activate p38 signaling and cause a transition from proliferation to quiescence. The cyclin D and Rb (retinoblastoma) pathways, which are important in liver development and regeneration, are affected by both events [127]. 

When the injurious agent acts persistently, disordered cell regeneration, inflammation, and fibrosis occur. In advanced stages, the normal population of hepatocytes is partially replaced by the disorganized deposition of these components of the extracellular matrix, which causes a decrease in hepatocellular mass and progressive anatomical and functional distortion of the liver [128]. One of the main causes of liver damage induced by several substances is oxidative stress. In this case, the hepatic regeneration capacity decreases, while the production of extracellular matrix components increases considerably [18]. Furthermore, supraphysiological concentrations of H_2_O_2_ can lead to hepatocyte growth arrest, cell death, and tissue pathology [129].

## 10. NOXs and Cancer

Oxidative stress plays a key role in many clinical phenomena, such as the inflammatory response and the aging process [130]. Recent studies have shown that oxidative stress is higher in many malignancies, including breast cancer, colon cancer, and head–neck neoplasms [131]. Oxidative stress is the major cause of enhanced cell migration, and it can induce the expression of oncogenes and suppress the activity of anti-survival molecules [132]. The level of oxidative stress is associated with the intracellular ROS level [133,134]. Disruption of coordinated NOX-derived ROS production is associated with carcinogenesis [135,136,137]. These oxidases are widely distributed in all cell membrane systems and their function depends on the type of tissue where the protein is expressed. It is important to note that not all NOXs are present in all cells and in all tissues. In some cases, only one type is found, such as in adipocytes that only have NOX4; however, some tissues or organs present more than two types of NOX, as is the case of the liver, which, in addition to presenting NOX2, presents NOX1, 4, and Duox1. The latter, as already mentioned, has to do with the function of the tissue, organ, cell type, and location of the cell within the organism [138]. 

### 10.1. NOX1

NOX1 are classical NOX associated with the cell plasma membrane (Figure 1), and they colocalize with caveolin, a scaffolding protein associated with caveolae, in punctuating patches of the surface in vascular smooth muscle cells (VSMCs) [139]. In hepatocytes, NOX1 activation requires the phosphorylation of the sarcoma kinase (Src) by TGF-β, which needs caveolin 1 and lipid raft domains [12]. NOX1, together with Src, mediates the activation of the tumor necrosis factor (TNF)-α-converting enzyme/a disintegrin and metalloproteinase 17 (TACE/ADAM17), and therefore, increases the shedding of different growth factors and cytokines, including EGFR ligands [140]. NOX1 produces ROS in endosomes after a hypoxia-reoxygenation injury, leading to c-Src activation after the recruitment of Rac-1 and c-Src; the c-Src-mediated activation of NF-κB is critical in the production of hepatic TNF-α [141,142]. It has also been reported that NOX1 is implicated in colon cancer, where its ROS-producing activity may enhance tumor cell proliferation and metastasis [143,144].

### 10.2. NOX2

**Figure 4:**
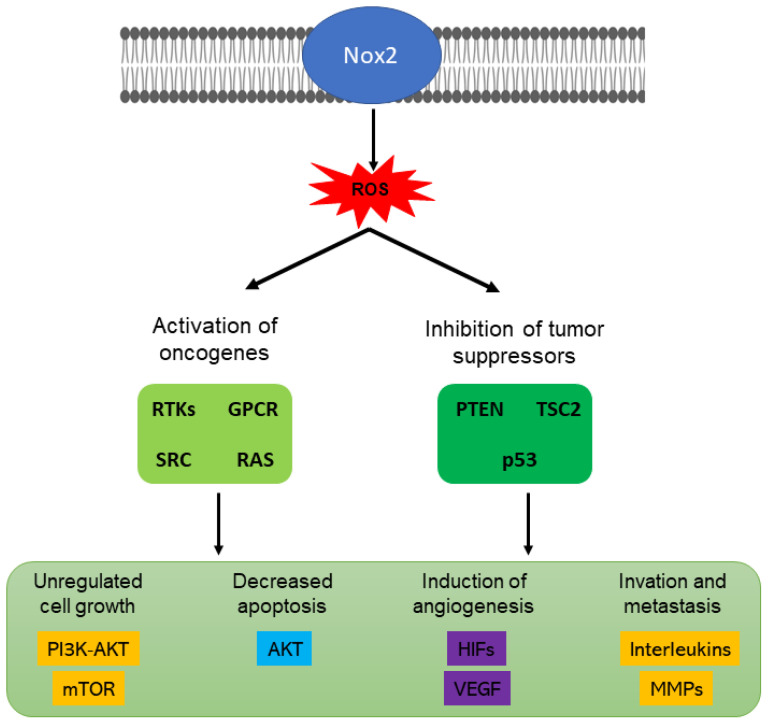
Signaling pathways possibly activated and inhibited by NOX2. Oncogene activation includes receptor tyrosine kinase (RTK), G protein-coupled receptors (GPCR), proto-oncogene tyrosine-protein kinase (SRC), and serine-threonine kinase (RAS). ROS inhibit tumor suppressors such as dual lipid tyrosine phosphatase (PTEN), tuberous sclerosis complex 2 (TSC2), and the p53 gene. All of these can affect different pathways to a greater or lesser extent, such as cell growth, decreased apoptosis, activation of angiogenesis, and invasion and metastasis. Other abbreviations: phosphoinositol 3-kinase (PI3K), target of rapamycin (mTOR), hypoxia-inducible factors (HIF), vascular endothelial growth factor (VEGF), and matrix metalloproteinases (MMPs). Modified from [135].

NOX2 was the first NOX isoform identified. It is expressed in phagocytic cells (Figure 2), on the lysosomal and plasma membranes of myeloid cells where it contributes to the phagocyte killing of microbes. NOX2 is minimally expressed by hematopoietic stem cells [145]. It plays an important role in cellular processes and can stimulate angiogenesis [134]. Additionally, NOX2 is an important effector of immune cell function, and its activity has been linked to oncogenic signaling [146]. NOX2 may stimulate tumor angiogenesis through vascular endothelial growth factor (VEGF). This, in turn, activates vascular endothelial growth factor receptor 2 (VEGFR2), a receptor tyrosine kinase (RTK) in endothelial cells to promote proliferation and migration. For this reason, VEGF represents a key molecule in the growth and metastasis of tumors [134] (Figure 4).

Myeloid leukemia cells express high levels of NOX2, which compromises the destruction of malignant cells by triggering ROS-induced apoptosis of adjacent antileukemic lymphocytes [147,148]. Stem cell expression of NOX2 has been implicated in leukemogenesis by maintaining the survival of leukemic stem cells [146]. Furthermore, the expression level of NOX2 was found to be increased in gastric cancer cells, where it promotes tumor progression [134,149]. On the other hand, an overactivation of NOX2 has been observed in patients with non-alcohol-associated fatty liver disease (NAFLD), related to an excess in the production of ROS and oxidative stress, which causes greater liver damage such as steatosis, inflammation, or fibrosis. Something similar occurs in patients with non-alcohol-associated steatohepatitis (NASH) who had significantly higher levels of NOX2-derivative peptide (sNox2-dp) [146]. It is important to note that NASH is emerging as one of the main causes of hepatocellular carcinoma (HCC) [150,151]. 

### 10.3. NOX4

NOX4, characterized by ubiquitous expression and continuous H_2_O_2_ production (Figure 1 and Figure 2), is the only isoform proposed to be constitutively active and negatively regulated by ATP [152]. The binding implies a generalized role for NOX4 in the maintenance of basal physiological redox homeostasis [153]. The NOX4 activity can be potentiated by hypoxia and consequently, the production of ROS, which can contribute to cancer malignancy. Likewise, hypoxia can instigate its mRNA transcription and/or protein translation [154,155]. Elevated levels of NOX4 protein and mRNA have been identified in cancers of various origins; an example of this is its increased expression in premalignant fibrotic states that can lead to lung and liver carcinomas [135,137,156,157]. 

Hypoxia-induced cellular responses are coordinated by the hypoxia-inducible transcription factors (HIFs) and the AMP-activated protein kinase (AMPK). Enhanced signaling by HIFs and AMPK has been identified in various tumors and linked to the cancerous rewiring of cellular metabolic processes [158]. NOX4 is a target gene of the hypoxia-sensitive transcription factor HIF-1. Conversely, NOX4-derived H_2_O_2_ is necessary for the hypoxia-related stabilization of HIF-1 [130].

### 10.4. DUOX1

Dual oxidase 1 (DUOX1) is predominantly found in the thyroid, which is involved in the synthesis of thyroid hormones [159] and its main function is the production of ROS [137] (Figure 1). It is also highly expressed in normal epithelial cells in the airway, pancreas, placenta, prostate, testis, and salivary gland [137,160]. Recent research indicates that DUOX1 may function as a selective tumor-suppressor gene (TSG) during tumor initiation and progression. DUOX1 is frequently silenced in lung cancer cells by its promoter hypermethylation [161]. In poorly differentiated follicular thyroid carcinoma, high expression of DUOX1 is associated with reduced risk of death [162]. Moreover, in 2014, Ling and collaborators found that DUOX1 expression is also frequently decreased in most liver cancer cell lines and primary hepatocellular carcinoma (HCC) tissues compared to its expression in non-tumor tissues. The silencing of DUOX1 gene expression is mediated by promoter hypermethylation and DUOX1 appears to be a functional tumor suppressor involved in liver carcinogenesis.

During malignant transformation and hepatic carcinogenesis, ROS are overwhelmingly produced, creating an oxidative microenvironment that can generate different and various types of cellular stress, including DNA damage, ER stress, cell death of damaged hepatocytes, as well as oxidative stress. Members of the NADPH oxidase family, such as NOX1, NOX2, and NOX4, have been clearly linked to the production of ROS in the liver [18], which may contribute to HCC development. Indeed, different NOX subunits, including p47^phox^, p67^phox^, and Rac1, were found to be increased in pre-neoplastic and neoplastic lesions [55].

In Table 1, we present NOXs as therapeutic targets of some drugs used so far that have given some positive results in the health of patients with the stated conditions, and which are the basis for the further development of more specific drugs to inhibit NOX without presenting serious side effects in patients.

## 11. Conclusions

As mentioned throughout this review, the production of ROS generated by NADPH oxidases has different functions, such as the regulation of the immune response, apoptosis, cell proliferation, etc. However, an increase in these oxidant molecules can generate or be linked to various conditions in living beings, especially in humans. Examples of these include fibrosis and cancer, but other pathologies linked to these proteins can occur, such as diabetes, and cardiovascular and neurodegenerative diseases. For this reason, researchers continue to develop more specific drugs that do not cause adverse effects (affecting other metabolic pathways or enzymes), and that allow these proteins to maintain adequate activity in the different physiological processes in which they are involved, in order to guarantee cellular homeostasis.

## Figures and Tables

**Table 1:** Some examples of drugs used to inhibit the activity of NOX in liver diseases.

Drug	Nox Inhibited	Pathway Affected	Disease	Study Model	Reference
LDC7559 (NA-11) (*Indirect action*)	NOX2	Respiratory burst (neutrophils)	Viral infection	Human blood	[163]
GKT137831 (Setanaxib) (*Direct action*)	NOX1/NOX4	Suppressed chemokine production, inhibited hepatic stellate cell (HSC) activation	Hepatic fibrosis	Mouse	[164]
GKT137831 (Setanaxib) (*Direct action*)	NOX1/NOX4	Bile duct ligation-induced hepatic fibrosis (BDL)	Hepatic fibrosis and hepatocyte apoptosis	Mouse	[77]
GKT137831 (Setanaxib) (*Direct action*)	NOX1/NOX4	Decrease in oxidative stress and inflammation	Hepatic fibrosis	Mouse	[165]
Chlorogenic acid (*Indirect action*)	NOX	Upregulation of NFE2L2, a transcription factor that regulates the expression of antioxidant enzymes	Hepatic fibrosis	Rats	[166]
Losartan (*Indirect action*)	Non-specific inhibition of different NOX	The expression of profibrogenic and NOX genes was reduced	Hypertension and heart failure	Human	[167]
Catalpol *(Indirect action*)	NOX4	AMPK/NOX4/PI3K/AKT	Hepatic insulin resistance in type 2 diabetes	Mouse	[168]
Apocinin (*Direct action*)	NOX2	Inhibits the binding of p47^phox^ to gp91^phox^	Inflammation and aging	Rats	[169]
Statinas (*Direct action*)	NOX1/NOX2	Inhibit Rac binding to gp91^phox^	Hepatic fibrosis	Rats	[170]

## Abbreviations

**ROS**	reactive oxygen species;
**HCC**	hepatic carcinogenesis;
**HBV**	hepatitis B virus;
**HCV**	hepatitis C virus;
**ALD**	alcohol-related liver disease;
**NAFLD**	nonalcoholic fatty liver disease;
**NADPH**	nicotinamide adenine dinucleotide phosphate hydrogen;
**KC**	Kupffer cells;
**HSCs**	hepatic stellate cells;
**ECs**	endothelial cells;
**PRR**	pattern recognition receptor;
**DAMPs**	damage-associated molecular patterns;
**PAMPs**	pathogen-associated molecular patterns;
**HAMPs**	homeostasis-altering molecular processes;
**NOD**	nucleotide-binding oligomerization domain;
**NLRC**	NOD-like receptor CARD domain containing;
**NLRP**	NOD-like receptor Pyrin domain containing;
**TNFR**	tumor necrosis factor receptor;
**TLR**	Toll-like receptors;
**ECM**	extracellular matrix;
**TGF-β1**	transforming growth factor β1;
**αSMA**	α-smooth muscle actin;
**BDL**	bile duct ligation;
**NASH**	non-alcohol associated steatohepatitis;
**HIF-1α**	Hypoxia Inducible Factor 1α;
**ET-1**	endothelin-1;
**LSEC**	liver sinusoidal endothelial cells;
**DAAs**	direct-acting antiviral agents;
**HCC**	hepatocellular carcinoma;
**SVR**	sustained virologic response;
**FXR or NR1H4**	farnesoid receptor;
**PKCf**	Protein kinase C f;
**ERK**	Extracellular signal-regulated kinase;
**VSMCs**	Vascular Smooth Muscle Cells;
**EGFR**	epidermal growth factor receptor;
**VEGF**	vascular endothelial growth factor;
**VEGFR2**	vascular endothelial growth factor receptor 2;
**RTK**	receptor tyrosine kinase;
**GPCR**	G protein-coupled receptors;
**SRC**	protooncogene tyrosine-protein kinase;
**RAS**	serine-threonine kinase;
**PTEN**	lipid tyrosine phosphatase;
**PI3K**	phosphoinositol 3-kinase;
**mTOR**	target of rapamycin;
**HIF**	hypoxia-inducible factors;
**VEGF**	vascular endothelial growth factor;
**MMPs**	matrix metalloproteinases;
**HIFs**	hypoxia-inducible transcription factors;
**AMPK**	AMP-activated protein kinase;
**TSG**	tumor-suppressor gene.
